# Exposure to varenicline protects against locomotor alteration in a MPTP mouse model of Parkinson's disease

**DOI:** 10.1590/1414-431X2021e11679

**Published:** 2021-12-03

**Authors:** A. Ribeiro-Carvalho, P.H. Leal-Rocha, J. Isnardo-Fernandes, U.C. Araújo, Y. Abreu-Villaça, C.C. Filgueiras, A.C. Manhães

**Affiliations:** 1 Departamento de Ciências, Faculdade de Formação de Professores, Universidade do Estado do Rio de Janeiro, São Gonçalo, RJ, Brasil; 2 Laboratório de Neurofisiologia, Departamento de Ciências Fisiológicas, Instituto de Biologia Roberto Alcântara Gomes, Centro Biomédico, Universidade do Estado do Rio de Janeiro, Rio de Janeiro, RJ, Brasil

**Keywords:** Parkinson's Disease, Dopamine, Neuroprotection, Varenicline, Nicotine

## Abstract

The beneficial effects of drugs that act via nicotinic acetylcholine receptors (nAChRs) on Parkinson's disease (PD) symptomatology may explain the negative correlation between cigarette smoking and risk of this neurological condition. Varenicline, an α4β2 nAChR partial agonist approved for smoking cessation treatments, could be valuable for PD treatment. Here, we investigated varenicline effects in a 1-methyl-4-phenyl-1,2,3,6-tetrahydropyridine (MPTP) PD mouse model. From postnatal day (PN) 90 to PN119, male C57BL/6 mice were exposed daily to varenicline (2 mg/kg) by gavage. After that, MPTP was injected (30 mg/kg, *ip*) once a day for five days. At PN125, locomotor and anxiety-like effects were assessed with the open field test. At PN126, immobile behavior was assessed with the forced swimming test. At PN127, the frontal cerebral cortex was collected to evaluate dopamine and DOPAC levels. To verify whether varenicline was protective during the MPTP insult, a separate group of MPTP animals received varenicline from PN90 to PN124. MPTP reduced cortical dopamine content and increased dopamine turnover. Those effects were not reversed by varenicline treatment. Interestingly, varenicline reversed the MPTP-induced hyperactivity in the open field. Both maintenance of varenicline treatment during MPTP exposure or its interruption before MPTP exposure elicited similar results. No alterations were observed in anxiety-like behavior or in immobility time. Altogether, these findings suggested that varenicline treatment reduced the MPTP-induced hyperactivity, but did not protect against dopaminergic damage. Based on this partial protective effect, varenicline could exert neuroprotective effects on circuits that control motor activity in PD.

## Introduction

It is well established in the literature that smoking is a protective factor against Parkinson's disease (PD) (1). Although cigarettes have a large number of psychoactive components that could affect several neurotransmitter systems (2), it has been proposed that the modulation of the cholinergic system is crucial to the pathophysiology of this disease. In fact, nicotine, the main psychoactive component of cigarettes, has been shown to protect against nigrostriatal damage in distinct animal models of PD, and several nicotinic cholinergic receptor subtypes (α4β2, α6β2, and α7) appear to be involved (3). In addition to its potential neuroprotective action, nicotine has antidepressant properties and enhances attention and cognition (4), which could contribute to the improvement of non-motor PD symptoms.

Varenicline is described as a highly efficient drug for smoking cessation therapy (5). It acts as a partial agonist of α4β2 and α6β2 nicotinic acetylcholine receptors (nAChRs) and a full agonist of the α7 receptor and increases dopamine release via neuronal nAChRs in the nucleus accumbens (6). Despite that, to date, only a few studies addressed the possibility that varenicline, similarly to nicotine, protects the dopaminergic system in PD. Varenicline prevents substantia nigra neurodegeneration induced by 6-hydroxydopamine as well as haloperidol-induced parkinsonism (7,8). As dopaminergic degeneration evoked by 1-methyl-4-phenyl-1,2,3,6-tetrahydropyridine (MPTP) exposure is widely used as a PD model in rodents, as well as a means to investigate the neuroprotective potential of compounds that modulate MPTP-induced effects, here, we investigated whether chronic varenicline has protective effects on behavior and the dopaminergic system in a MPTP mice model.

## Material and Methods

All procedures were approved by the Institute of Biology/UERJ Ethical Committee for Animal Research (Protocol CEUA/015.14). All C57BL/6 mice were bred and maintained in our laboratory vivarium at 21-22°C on a 12-h light/dark cycle (lights on at 2:00 a.m.). Access to food and water was *ad libitum*.

For 30 days, from postnatal day (PN) 90 to PN119, adult male mice were subcutaneously treated daily with saline (0.9%, w/v NaCl) or varenicline (2 mg/kg) solution. This dose of varenicline is within the range used in rodent studies (8,9). After that, animals were injected with MPTP (30 mg/kg body mass, *ip*) or saline once daily for five days (PN120-124). Accordingly, mice from each litter were distributed into four treatment groups: CONT (saline injections), MPTP (saline + MPTP), VAR (2 mg/kg of varenicline + saline), and VAR+MPTP (2 mg/kg of varenicline + MPTP). In addition, to verify whether varenicline would be specifically protective if administered during the MPTP insult, in a separate group of MPTP animals, varenicline treatment was maintained during the entire MPTP exposure period, from PN90 to PN124 (VARext+MPTP).

### Behavior evaluations

All behavioral tests were carried out in a sound-attenuated room next to the animal facility. At PN125, we evaluated locomotor activity and anxiety-like behavior with the open field (OF) test (8:00 to 10:00 a.m.). At PN126, the immobile behavior was assessed in the forced swimming test (FST, 2:00 to 4:00 p.m.).

### Open field

The OF arena consists of a plastic container (37.6×30.4 cm) surrounded by 17-cm-high walls with the floor divided into 16 rectangles of equal area (inner area = 7.6×9.4 cm). The test was continuously recorded for 10 min by an overhead video camera. The total distance traveled was quantified based on the number of rectangles crossed (i.e., all four legs entering on a given square counted as a crossing) by an experimenter blind to the animal's group and the observed value was used as a measure of locomotor activity. The percent of time spent in the center squares (%Time in center = time spent in the center/total time) was used as a measure of anxiety-like behavior. At the end of the session, the OF arena was thoroughly cleaned with ethanol solution (30%) and dried.

### Forced swimming test

Briefly, each mouse was placed in a plastic container (diameter, 21 cm; height, 23 cm) filled with 16 cm of water at about 25°C. The animal's behavior was continuously recorded throughout the testing session (6 min) with an overhead video camera. Animals were considered to be immobile when they remained floating with all limbs and tail motionless. The time the animals spent in this condition was used as a measure of depressive-like behavior.

### DA and DOPAC evaluations

Mice were decapitated between 2:00 and 6:00 p.m. at PN127. The cerebral cortex was separated from the midbrain/brain stem by a cut made caudal to the thalamus. The frontal cerebral cortex, corresponding to the anterior 1/3 of the hemispheres, was collected. The tissue was weighed, frozen in liquid nitrogen, and stored at -45°C until assay. Dopamine (DA) and 3,4 dihydroxyphenylacetic acid (DOPAC) levels were analyzed in the right frontal cerebral cortex by high-performance liquid chromatography (HPLC) using a fluorescence detector (RF-20A detector, Shimadzu Prominence LC-20AT, Japan). Briefly, each cortex was thawed and homogenized with 200 μL of HClO_4_ (0.1 M) plus sodium ascorbate (20 μM) (Precellys, BERTIN Technologies, France) and centrifuged at 5,200 *g* for 30 min (4°C). The supernatant was filtrated in a 0.22-μm PVDF filter (Millipore, USA) and diluted in 4 volumes of milli-Q water. DA and DOPAC derivatization reaction was accomplished using 10 μL standard solution or tissue sample + 20 μL of glycine (2.5 M) + 10 μL NaIO_4_ (5 mM) + 20 μL of acetonitrile + 50 μL of derivatization solution (0.1 M 1,2-diphenyl-ethylenediamine/0.1 M glycine/62 mM sodium methoxide/3.75 mM potassium hexacyanoferrate III). After 3 min at ambient temperature, a 20-μL portion of the final reaction mixture was injected onto the chromatograph. The detector wavelengths of excitation/emission were 345/480 nm. The mobile phase was a gradient formed by acetonitrile and acetate buffer (20 mM, pH=4.5) with EDTA (0.5 mM). We used a 0.1 mL/min flow rate and ambient temperature (20-23°C). A reverse-phase column (150×2.6 mm i.d., packed with C 18 silica, 3 μm) was used for separation.

### Statistical analysis

Statistical analyses were performed using SPSS 20 software (IBM, USA). Univariate or repeated measures analyses of variance (ANOVAs) were used as appropriate. Group (CONT, VAR, MPTP, and VAR+MPTP) was considered the between-subjects factor. Fisher protected least significant difference (FPLSD) was used as the *post hoc* test. Separate ANOVAs were performed to compare the groups VAR+MPTP and VARext-MPTP. All data are reported as means±SE. Significance was assumed at the level of P<0.05.

## Results

Varenicline and MPTP treatments did not affect body mass gain (Supplementary Table S1).

**Figure 1:**
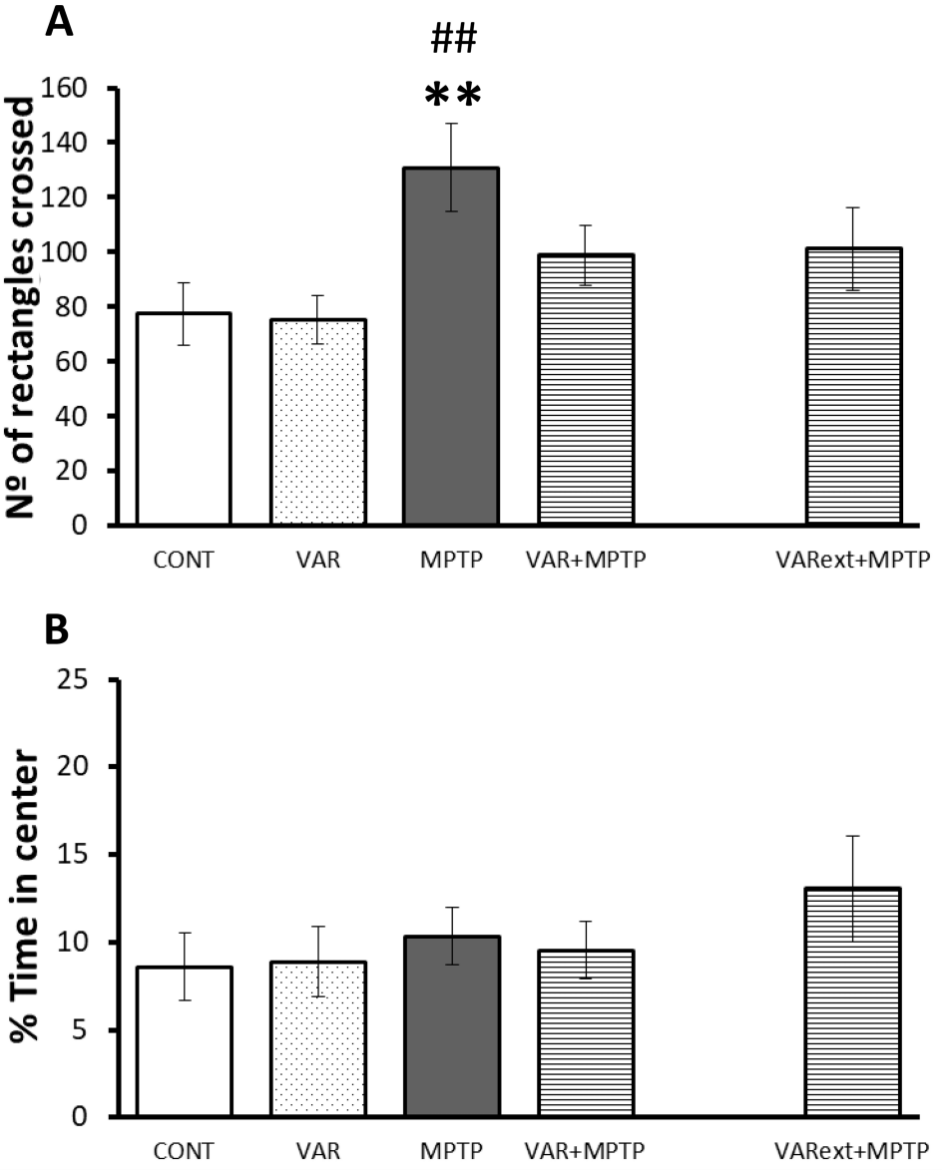
Effects of chronic varenicline exposure on behavior of mice in the open field test in a 1-methyl-4-phenyl-1,2,3,6-tetrahydropyridine (MPTP) mouse model of Parkinson's disease. Locomotor activity quantified based on the number of rectangles crossed (**A**) and anxiety levels (**B**) evaluated by percent of time spent in the center of arena. Data are reported as means±SE. **P<0.01 *vs* control; ^##^P<0.01 *vs* VAR (ANOVA followed by Fisher protected least significant difference). CONT: saline injections; MPTP: saline + MPTP; VAR: 2 mg/kg of varenicline + saline; VAR+MPTP: 2 mg/kg of varenicline + MPTP; VARext+MPTP: varenicline treatment during MPTP exposure.

A significant effect of Group (F_(3,35)_=3.7; P=0.02) was identified regarding locomotor activity in the OF: MPTP animals showed increased locomotor activity in the OF test compared to CONT. While varenicline treatment *per se* did not affect locomotor activity, it reversed the MPTP hyperlocomotor effect (Figure 1A). No difference was observed between VAR+MPTP and VARext+MPTP. There were no differences in anxiety-like behavior, as indicated by percent time spent in the center of the arena (Figure 1B). No Group effects were observed for immobile behavior in forced swimming test (Table 1).

**Table 1:** Effect of chronic varenicline exposure on forced swimming test results.

	Immobility time (s)
CONT	49.8±9.5
VAR	46.5±11.2
MPTP	52.3±3.3
VAR+MPTP	38.0±5.8
VARext+MPTP	38.4±7.7

Data are reported as means±SE. There were no statistically significant differences (ANOVA). CONT: saline injections; MPTP (1-methyl-4-phenyl-1,2,3,6-tetrahydropyridine): saline + MPTP; VAR: 2 mg/kg of varenicline + saline; VAR+MPTP: 2 mg/kg of varenicline + MPTP; VARext+MPTP: varenicline treatment during MPTP exposure.

**Figure 2:**
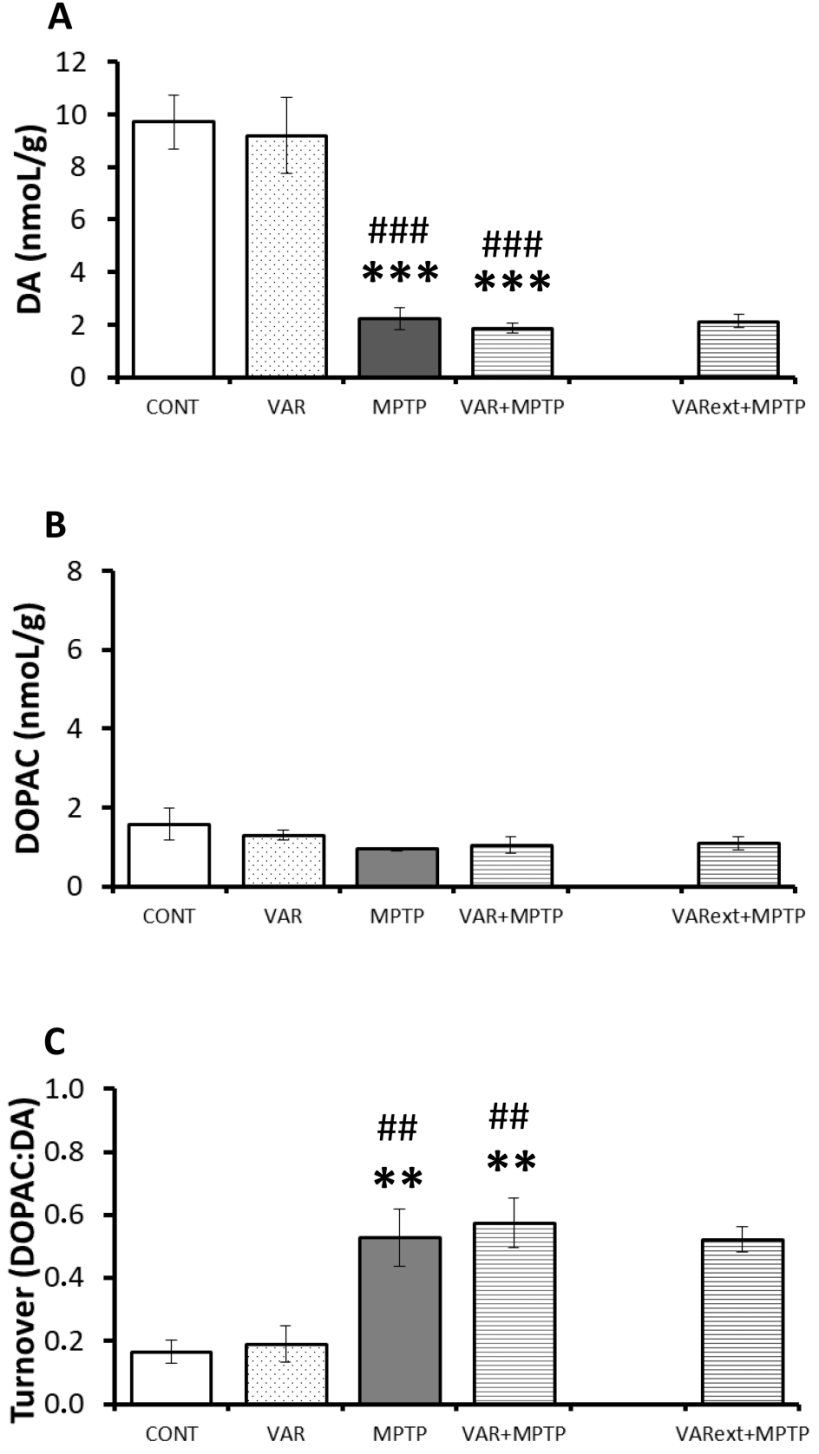
Effects of chronic varenicline exposure on dopamine (DA) levels (**A**), 3,4 dihydroxyphenylacetic acid (DOPAC) levels (**B**), and turnover (**C**) in a 1-methyl-4-phenyl-1,2,3,6-tetrahydropyridine (MPTP) mouse model of Parkinson's disease. Data are reported as means±SE. **P<0.01, ***P<0.001 *vs* control; ^##^P<0.01, ^###^P<0.001 *vs* VAR (ANOVA followed by Fisher protected least significant difference). CONT: saline injections; MPTP: saline + MPTP; VAR: 2 mg/kg of varenicline + saline; VAR+MPTP: 2 mg/kg of varenicline + MPTP; VARext+MPTP: varenicline treatment during MPTP exposure.

A Group effect was observed regarding DA levels (F_(3,35)_=32.3; P<0.001): MPTP exposure decreased DA content in the frontal cerebral cortex, as expected; varenicline treatment did not affect this outcome (Figure 2A). Varenicline treatment on its own did not affect DA levels. DOPAC levels were not affected (Figure 2B), but a significant effect of Group was observed regarding the DOPAC:DA ratio (F_(3,35)_=9.9; P<0.001). It was similarly increased in both MPTP-exposed groups (MPTP and VAR+MPTP) compared to the CONT and VAR groups, suggesting an increase in dopamine turnover (Figure 2C). Varenicline treatment *per se* did not affect DOPAC:DA ratio or alter MPTP-induced increase in dopamine turnover. In all biochemical measures, no differences were observed between VAR+MPTP and VARext+MPTP groups.

## Discussion

In the present study, we evaluated potential neuroprotective effects of varenicline in an animal model of PD. Varenicline is a drug that acts as a partial agonist of α4β2 and α6β2 cholinergic receptors, and total agonist of α7 receptors. It has been used as an efficient pharmacological approach in anti-smoking therapy, with great tolerance even when used for long periods (10). Here, the PD model of subchronic exposure to MPTP was able to reduce dopamine content in the frontal cortex by approximately 75%, which is consistent with previous data (11). This effect probably generated compensatory mechanisms, increasing dopamine turnover and inducing hyperactivity in the OF test. Despite the fact that the varenicline treatment was not able to protect against dopaminergic or DOPAC:DA ratio changes in mice exposed to MPTP, it was able to prevent the manifestation of behavioral hyperactivity. Similar behavioral and neurochemical effects were identified both when varenicline treatment was ended before MPTP exposure and when it was extended to occur concomitantly to the MPTP insult. These findings indicated that varenicline effects were mainly neuroprotective and not mediated by a direct interference with MTPT toxic effects.

The MPTP-induced spontaneous hyperactivity in the OF is frequently described, particularly when subchronic dose regimens are used (12). Our hypothesis considered that the hyperactivity response was associated with an adaptive response of the injured motor pathways, generated by the change in activity of the surviving dopaminergic neurons. We observed an increase in the DOPAC:DA ratio in animals exposed to MPTP. This finding indicated an increase in dopamine turnover and seemed to be related to a compensatory response to the reduction in dopaminergic terminals (13).

The present study showed that varenicline protected against MPTP-induced locomotor hyperactivity. Similar effects have been observed recently in other models of hyperactivity. Recently, it has been reported that varenicline reduces motor hyperactivity generated by ethanol and tends to attenuate the expression of ethanol-induced sensitization (14). In addition, a report indicates that the use of varenicline reduces symptoms of attention deficit hyperactivity disorder (15). The protection against MPTP-induced locomotor hyperactivity could be attributed to the protection of dopaminergic neurons via increased α4β2* nAChR expression, as this subtype constitutes a major component of the neuroprotective effect of nicotine (16). Here, varenicline did not protect against changes in cortical DA levels in animals exposed to MPTP; however varenicline effects may not be limited to the α4β2*-nAChR. Post-mortem analysis indicates that PD patients express significantly less α7-nAChRs in the temporal cortices (17). In this sense, it is possible to speculate that the α7-nAChR upregulation induced by chronic varenicline treatment could attack some symptoms of PD. Some evidence also shows that activation of α7 receptors can alleviate PD symptoms in animal models (18). Crunelle et al. (9) showed that a two-week treatment with varenicline (2 mg·kg^-1^·day^-1^) significantly increases striatal dopamine receptors availability in rodents and may play a role in the effects observed in the present study (9). In accordance, Sharma et al. (8) demonstrated that haloperidol-induced Parkinsonism is attenuated by varenicline in mice. In the same direction, varenicline improved motor deficits induced by 6-OHDA administration in rats (7). Additionally, administration of varenicline is associated with the reduction of dyskinesias induced by L-DOPA treatment in a PD model in rodents (19).

Studies have shown that non-motor manifestations of PD, such as mood disorders and cognitive deficits, are frequent in PD patients, which reduce their quality of life (20). In addition, non-motor symptoms in PD can sometimes appear many years before motor signs (20). Thus, it seems relevant to evaluate models of PD that mimic non-motor stages of the disease. Here, neither our MPTP model nor the varenicline treatment altered anxiety and depressive-like behavior. Future studies using chronic models of dopaminergic injury, where the damage occurs more slowly and gradually, could elucidate the role of varenicline on changes in emotional functions.

In conclusion, our data suggested that varenicline may be useful in the management of hyperactivity disorders. In addition, it could also exert neuroprotective and regulatory effects on circuits that control motor activity in PD. Future pre-clinical studies are warranted if one is to consider varenicline administration as a possible therapeutic agent for PD management. Other experiments focused on potential neurochemical markers such as alpha-synuclein accumulation, oxidative stress, and inflammatory response are essential to examine how varenicline generates its effects on Parkinson's disease.

## Supplementary Material

Click to view [pdf].
